# Health care utilization of patients with acute abdominal pain before and after emergency department visits

**DOI:** 10.1186/s13049-024-01237-7

**Published:** 2024-08-12

**Authors:** Katharina Verleger, Antje Fischer-Rosinsky, Martin Möckel, Anna Schneider, Anna Slagman, Thomas Keil, Liane Schenk, the INDEED Research Group, Natalie Baier, Reinhard Busse, Dominik Brammen, Johannes Drepper, Patrik Dröge, Felix Greiner, Cornelia Henschke, Stella Kuhlmann, Björn Kreye, Christian Lüpkes, Thomas Reinhold, Burgi Riens, Marie-Luise Rosenbusch, Felix Staeps, Kristin Schmieder, Daniel Schreiber, Dominik von Stillfried, Maike Below, Rainer Röhrig, Stephanie Roll, Thomas Ruhnke, Felix Walcher, Grit Zimmermann, Ryan King

**Affiliations:** 1 Institute of Medical Sociology and Rehabilitation Science, Charité – Universitätsmedizin Berlin, Berlin, Germany; 2 Department of Emergency and Acute Medicine, Charité – Universitätsmedizin Berlin, Berlin, Germany; 3 Institute of Social Medicine, Epidemiology and Health Economics, Charité – Universitätsmedizin Berlin, Berlin, Germany; 4 Institute of Clinical Epidemiology and Biometry, University of Würzburg, Würzburg, Germany; 5 State Institute of Health I, Bavarian Health and Food Safety Authority, Erlangen, Germany

**Keywords:** Emergency care, Abdominal pain, Routine health care data, Cross-sectoral health care

## Abstract

**Background:**

Acute abdominal pain (AAP) is a major driver for capacity-use in emergency departments (EDs) worldwide. Yet, the health care utilization of patients with AAP before and after the ED remains unclear. The primary objective of this study was to describe adult patients presenting to the ED with AAP and their outpatient care (OC) use before and after the ED. Secondary objectives included description of hospitalization rates, in-hospital mortality, ED re-visits, and exploration of potential risk factors for hospitalization and ED re-visits.

**Methods:**

For the analysis, we combined routine hospital data from patients who visited 15 EDs in Germany in 2016 with their statutory health insurance OC claims data from 2014 to 2017. Adult patients were included based on a chief complaint or an ED diagnosis indicating unspecific AAP or the Manchester Triage System indicator “Abdominal pain in adults”. Baseline characteristics, ED diagnosis, frequency and reason of hospitalization, frequency and type of prior-OC (prOC) use up to 3 days before and of post-OC use up to 30 days after the ED visit.

**Main results:**

We identified 28,085 adults aged ≥ 20 years with AAP. 39.8% were hospitalized, 33.9% sought prOC before the ED visit (48.6% of them were hospitalized) and 62.7% sought post-OC up to 30 days after the ED visit. Hospitalization was significantly more likely for elderly patients (aged 65 and above vs. younger; adjusted OR 3.05 [95% CI 2.87; 3.25]), prOC users (1.71 [1.61; 1.90]) and men (1.44 [1.37; 1.52]). In-hospital mortality rate was 3.1% overall. Re-visiting the ED within 30 days was more likely for elderly patients (1.32 [1.13; 1.55) and less likely for those with prOC use (0.37 [0.31; 0.44]).

**Conclusions:**

prOC use was associated with more frequent hospitalizations but fewer ED re-visits. ED visits by prOC patients without subsequent hospitalization may indicate difficulties of OC resources to meet the complex diagnostic requirements and expectations of this patient population. Fewer ED re-visits in prOC users indicate effective care in this subgroup.

**Supplementary Information:**

The online version contains supplementary material available at 10.1186/s13049-024-01237-7.

## Background

Acute abdominal pain (AAP)—defined as atraumatic abdominal pain with a maximum duration of 7 days—accounts for 6–20% of all visits to emergency departments (ED) in Western Europe [1–4] It is associated with high in-hospital mortality (5.1% compared to 0.6% among patients with chest pain [3]), substantial long-term burden [5], and frequent ED re-visits [1, 6]. Still, AAP is often dismissed as trivial. AAP may be due to a variety of underlying diagnoses, for some of which ED treatment is not the ideal approach as they may need follow-up and/or regular check-up [7]. While it is often assumed that patients with AAP swing back and forth between different types of primary, secondary and tertiary health care, little is known of the exact treatment paths of patients before and after their ED visit. Sectoral barriers between outpatient primary and secondary care (OC) and hospital care are typically high in Germany, e.g., in terms of informational flow. This makes it difficult to bridge the gap between procedures necessary for the diagnosis of AAP and follow-up treatment in OC. This is particularly true for procedures such as sonography and computed tomography (CT), which are—as in many health care systems worldwide—more readily available in hospitals through the ED than in OC care settings. Thus, a better knowledge of patient pathways is necessary to ensure (more) appropriate and efficient patient care [8].

Up to 2.1 million patients with AAP are estimated to be treated in Germany’s EDs each year [3, 9]. Given the fact that AAP is not only very common but also causes the highest costs among all chief complaints at the ED, more efficient patient pathways would have a major impact on ED resources and budgets [10, 11]. Despite the heterogeneity of EDs, characterizations of patients with AAP, as well as diagnostic and treatment decisions, are mostly informed by single-center studies [1, 2, 6, 7, 12–14]. Since a better understanding of the patient profile of AAP in emergency care is needed to improve patient care, outcomes and resource allocation, generalizable real-world data need to be analyzed on a nationally representative level. Therefore, this analysis was based on data from the INDEED study (Utilization and cross-sectoral patterns of care for patients admitted to emergency departments in Germany) [15], which collected data on all patients treated in 16 structurally different German EDs, their subsequent hospital stay, and their OC before and after the ED visit. The primary objective of this study was to describe the adult patient population presenting to the ED with AAP in Germany and their OC use before and after the ED. Secondary objectives included hospitalization rates, in-hospital mortality, and ED re-visits, as well as the exploration of risk factors.

## Methods

### Study design and variables

INDEED was a large-scale database study funded by the Innovation Fund of the German Joint Federal Committee (01NVF19025). It combined routine data from 16 EDs in Germany from 2016 and OC claims data of these ED patients from 2014 to 2017. Further study details including variables, data management and protection measures as well as ethical aspects were published by Fischer-Rosinsky et al. [15]. One ED was excluded from this study since it did not provide data on non-hospitalized patients.

Routine patient data from the ED and the OC sector were linked using a unique patient identifier. Sample characteristics (sex, age, ED district type, Manchester Triage System [MTS] status) and ED/hospital care characteristics (ED date and time of visit, ED diagnosis [patients could receive multiple diagnoses at the same visit], hospitalization, main hospital diagnosis, data of OC utilization before and after ED visit, and recurrent ED visits [re-visits] within 30 days) were analyzed. Elderly patients were defined as being ≥ 65 years of age at ED visit. Participating EDs were categorized into district types (urban vs. rural areas) according to the Federal Office for Building and Regional Planning (BBSR) and modified by the number of EDs in the area [16]. If there was not more than one ED in the municipality, an area was categorized as “rural”. MTS status was categorized as urgent (i.e., triage category red, orange or yellow) or non-urgent (i.e., green or blue) [13]. To examine cross-sectoral patterns of care, which may be acutely related to the cause of the ED visit, the number and type of OC visits were determined 3 days prior (“prOC”), and 30 days after the ED visit (“post-OC”). These time periods were defined in line with previous studies and clinical advice: patients must have been able to—at least theoretically—seek outpatient primary care outside of weekends (thus 3 days including a weekday) [1, 6]. The dates of all OC visits were based on physicians’ tariffs claims data [17]. OC visits on the same day as the ED visit were considered to occur before the ED visit. Data from laboratories, as well as any tariffs indicating lab analysis, were excluded because a lab analysis claim may refer to the date that the analysis was performed, not to the day the sample was collected/an OC visit was made. If a patient visited both a general practitioner (GP) and a specialist, only the utilization of the specialist was included in the analysis presuming that the patient was referred to the specialist by the GP, as is the typical procedure in Germany.

### Inclusion criteria

In the INDEED-AAP dataset, analyses were performed on adult patients (≥ 20 years) with statutory health insurance meeting one or more of the following inclusion criteria: (i) chief complaint indicating AAP, including upper AAP, lower AAP, flank pain, and stomach pain; (ii) any ED diagnosis indicating unspecific AAP in line with the International Classification of Diseases (ICD) category R10 (Abdominal and pelvic pain) at the ED visit; (iii) MTS indicator “abdominal pain in adults”. Patients were excluded from analysis if the date of the ED visit was missing, since their prOC and post-OC use could not be determined.

### Statistical analyses

Categorical variables were summarized using counts and percentages; continuous variables using the mean, median, 95% standard deviation (SD) and range. Chi-squared, Fisher’s exact, and Student’s t-tests were used to determine univariate associations between dichotomized sample characteristics: elderly (aged 65 + years) vs. non-elderly (< 65 years); men vs women; rural vs urban; prOC users (patients who used OC 3 days before their ED visit) vs non-prOC users pre-ED (patients who did not seek OC within 3 days before their ED visit). Odds ratios (ORs) were computed with corresponding 95% confidence intervals (CIs). Missing data were reported. Subsequently, a multivariable logistic regression was conducted to examine risk factors using a set of variables that was defined a priori. As sensitivity analyses, a fixed-effects multilevel model was used to account for clustering within clinics. Data analyses were conducted using SAS version 9.4. Sankey diagrams were re-formatted using sankeyMATIC.

## Results

**Fig. 1:**
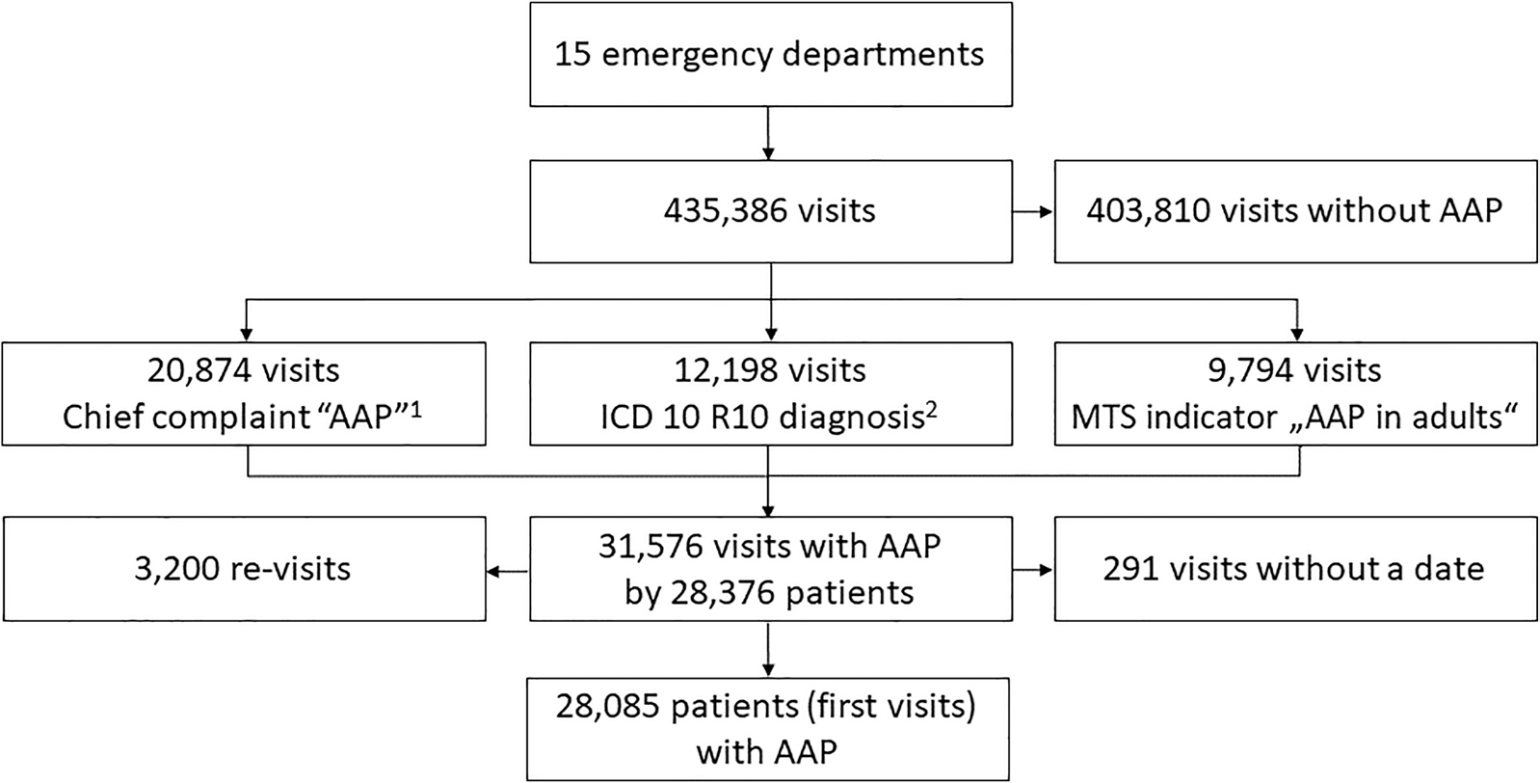
Flow chart of patients. *AAP* acute abdominal pain; *ICD* international classification of disease; *MTS* Manchester triage system; *R10* abdominal and pelvic pain. ^1^For 3,904 of these patients, an additional ICD 10 diagnosis R10 was recorded, for 3,384 patients, the MTS indicator “AAP in adults” was recorded, and for 1,348 all three categories were recorded. ^2^For 1,306 of these patients an additional MTS indicator “AAP in adults” was recorded

In 15 participating EDs with 435,386 visits in total, 31,576 visits were identified (7.3%) by 28,376 patients with AAP. After exclusion of 291 patients (1.0%) with missing date of the ED visit, the final sample included 28,085 patients (Fig. 1).

### Sample characteristics

**Fig. 2:**
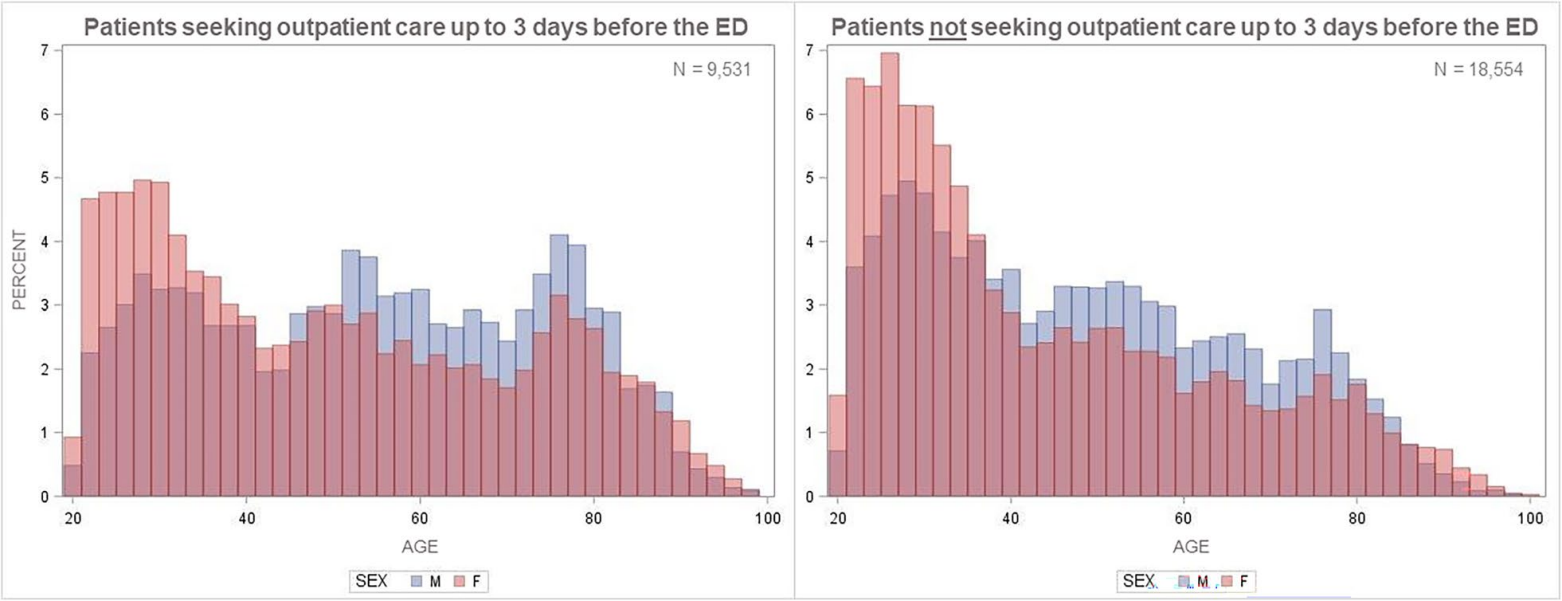
Histogram of patient's age, by sex and outpatient care use before the ED. The graphs shows the distribution of age (in 2-year categories) and sex (blue = men, red = women), separately for patients who sought outpatient care up to 3 days before their emergency department visit (left) and patients who did not utilize outpatient care (right)

Table 1 shows sample characteristics, differentiated by prOC utilization. An extended version of Table 1 differentiating by age group and sex is available as an additional file. The patients’ mean age was 47.7 years (SD 19.9, Fig. 2); a higher proportion of patients (58.3%) were women (Table 1). The majority of all patients (22,404; 79.9%) were treated in one of the nine EDs in an urban area.

**Table 1:** Sociodemographic, medical and outpatient care characteristics of ED patients with acute abdominal pain

	All (N = 28,085)	prOC user (N = 9531; 33.6%)	Non-prOC user (N = 18,554; 65.4%)
Age, mean (SD)	47.7 (19.9)	51.6 (20.6)	45.7 (19.3)
Female, N (%)	16,375 (58.3)	5,803 (60.9)	10,572 (57.0)
Urban area, N (%)	22,404 (79.8)	7,716 (81.0)	14,688 (79.2)
MTS status “urgent”, N (%)	15,905 (56.6)	5,317 (55.8)	10,588 (57.1)
Missing, N (%)	2,978 (10.6)	1,129 (11.8)	1,849 (10.0)
prOC use, N (%)	9,531 (33.9)	9,531 (100)	0 (0.0)
Type of prOC provider, N (%)			
General practitioner	5,293 (18.8)	5,293 (55.5)	0 (0.0)
Specialist	4,003 (14.3)	4,003 (42.0)	0 (0.0)
Not documented	235 (0.8)	235 (2.5)	0 (0.0)
Hospitalized, N (% of total N)	11166 (39.8)	4,632 (48.6)	6,534 (35.2)
ICD-10 hospital diagnoses			
Missing, N (% of hospitalized)	133 (1.2)	98 (2.1)	35 (0.5)
Top1 (% of total)	R10 (3.4)	R10 (4.5)	R10 (2.7)
Top2 (% of total)	K80 (2.6)	K80 (3.0)	K35 (2.5)
Top3 (% of total)	K35 (2.4)	K56 (2.7)	K80 (2.4)
Top4 (% of total)	K56 (2.1)	K35 (2.4)	K56 (1.8)
Top5 (% of total)	K57 (1.8)	K57 (2.4)	K85 (1.7)
In-hospital mortality N (% of hospitalized)			
Death reported	349 (3.1)	170 (3.7)	179 (2.7)
Missing	530 (4.8)	382 (8.2)	148 (2.3)
Post-OC use, N (%)	17,603 (62.7)	7,826 (82.1)	9,777 (52.7)
Type of post-OC provider, N (%)			
General practitioner	6,799 (24.2)	2,783 (29.2)	4,016 (21.6)
Specialist	10781 (38.4)	5,033 (52.8)	5,748 (31.0)
Not documented	23 (< 0.1)	10 (0.1)	13 (0.1)
Post-OC use after hospital, N (% of hospitalized)	7,451 (66.7)	3,786 (81.7)	3,665 (56.1)
Type of post-OC provider after hospital, N (% of hospitalized))			
General practitioner	3,448 (30.9)	1,637 (35.3)	1,811 (27.7)
Specialist	3,989 (35.7)	2,142 (46.2)	1,847 (28.3)
Not documented	14 (0.1)	7 (0.2)	7 (0.1)
ED re-visit in 30 days	1,101 (3.9)	181 (1.9)	920 (5.0)

*ED* emergency department; *ICD* international classification of disease; *N* number; *N10* acute tubulo-interstitial nephritis; *N13* obstructive and reflux uropathy; *OC* outpatient care; *post-OC* post-outpatient care (up to 30 days after ED visit); *prOC* prior outpatient care (up to 3 days before ED visit); *R10* abdominal and pelvic pain; *K35* acute appendicitis; *K56* paralytic ileus and intestinal obstruction without hernia; *K57* diverticular disease of intestine; *K80* cholelithiasis; *K85* acute pancreatitis

Of the 28,085 patients, 33.9% used prOC up to 3 days before their ED visit, with 14.1% seeking specialist care (Table 1). After the ED visit, 62.7% used post-OC and 3.9% re-visited the ED within 30 days. Use of prOC/post-OC was significantly more frequent among women (35.8%/65.9%) and elderly patients (44.1%/69.4%) than among men (31.8%/57.5%) and non-elderly patients (31.4%/60.3%). In-hospital mortality rate was 3.1% (N = 354), with significant increases for elderly patients (3.8%) and prOC users (3.7%, Table 1). The percentage of patients who re-visited the ED was significantly lower in prOC users versus non-prOC users (Table 1;).

### ICD-10 diagnoses

**Fig. 3:**
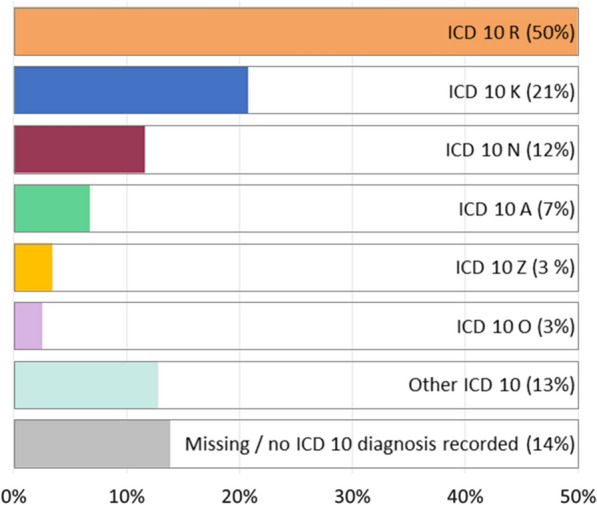
ICD 10 ED diagnoses of 16,919 non-hospitalized patients with AAP. *AAP* Acute abdominal pain; *ED* emergency department; *ICD* international classification of disease; *ICD 10 A* infectious and parasitic diseases; *ICD K* diseases of the digestive system; *ICD N* diseases of the genitourinary system; *ICD O* pregnancy, childbirth and the puerperium; *ICD R* symptoms, signs and abnormal clinical and laboratory findings, *ICD 10 Z* Factors influencing health status and contact with health services

**Fig. 4:**
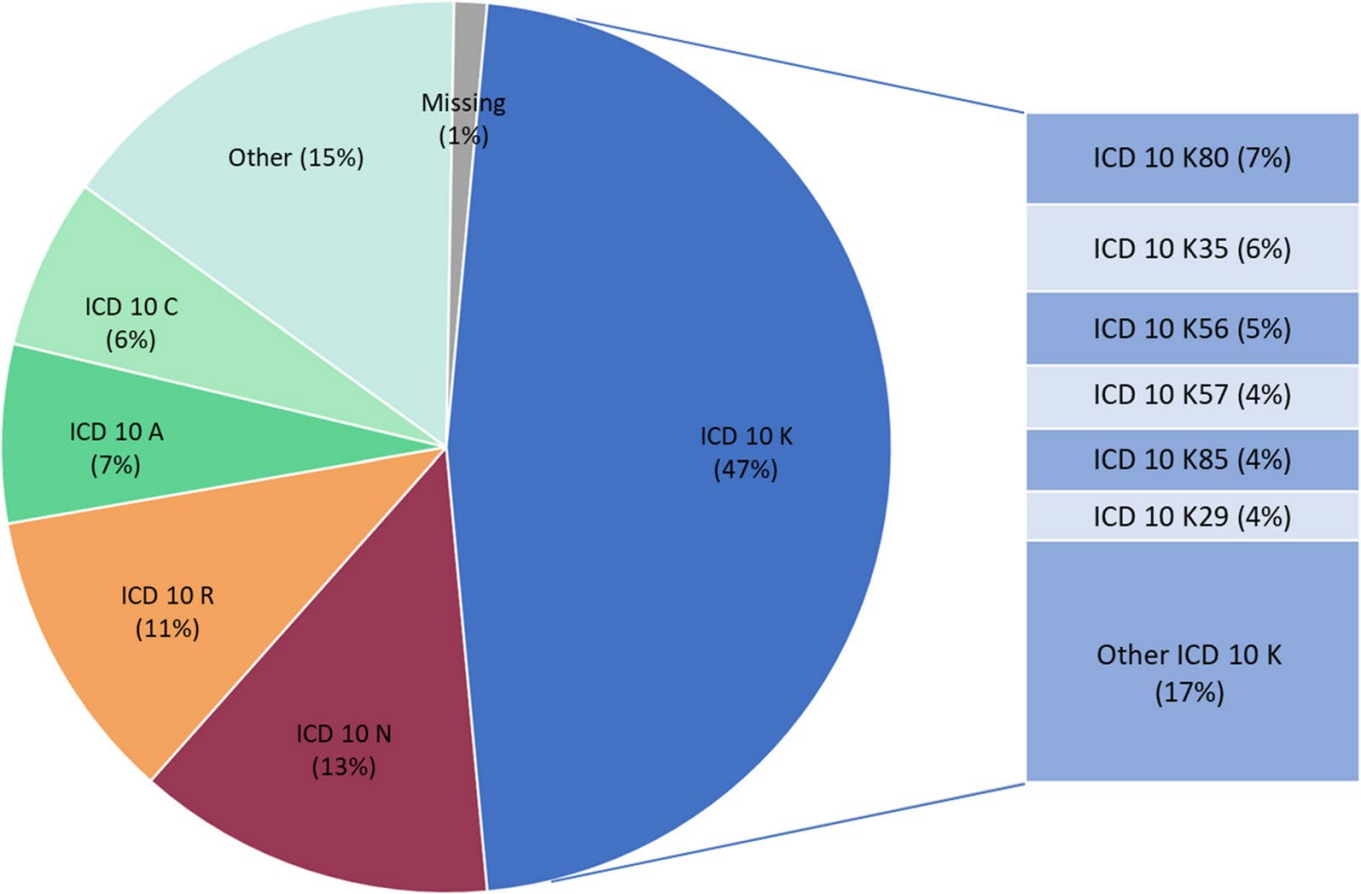
ICD 10 hospital diagnoses of 11,166 hospitalized patients with AAP. *AAP* acute abdominal pain; *ED* emergency department; *ICD* international classification of disease; *ICD 10 A* infectious and parasitic diseases; *ICD 10 C* neoplasms; *ICD K* diseases of the digestive system; *ICD N* diseases of the genitourinary system; *ICD R* symptoms, signs and abnormal clinical and laboratory findings. The category “Other” includes all other ICD 10 diagnoses chapters, each with a frequency of less than 3.8% of all hospitalized patients

An ED diagnosis was reported in 86.1% (N = 14,570) of 16,919 non-hospitalized ED patients with AAP. For 21.8% of patients (N = 3686), more than one ED diagnosis was reported. In line with inclusion criteria, ‘abdominal and pelvic pain’ (ICD-10 R10) was the most common diagnosis (Fig. 3). A hospital diagnosis was reported for 98.8% (N = 11,033) of 11,166 hospitalized patients, with “Diseases of the digestive system” being the most common ICD 10 diagnosis chapter (Fig. 4). The top 5 specific ICD 10 diagnoses (Table 1) explained only a minority of cases, reflecting the heterogeneity of causes for AAP as well as the commonness of symptom-based diagnoses in our study sample. The hospital diagnoses with the highest in-hospital mortality were ‘Sepsis’ (A41; N = 29 deaths), ‘Acute vascular disorders of the intestine’ (K55; N = 18), ‘Malignant neoplasm of the pancreas’ (C25; N = 17), and ‘Paralytic ileus and intestinal obstruction’ (K56; N = 14).

### Treatment patterns of ED patients

**Fig. 5:**
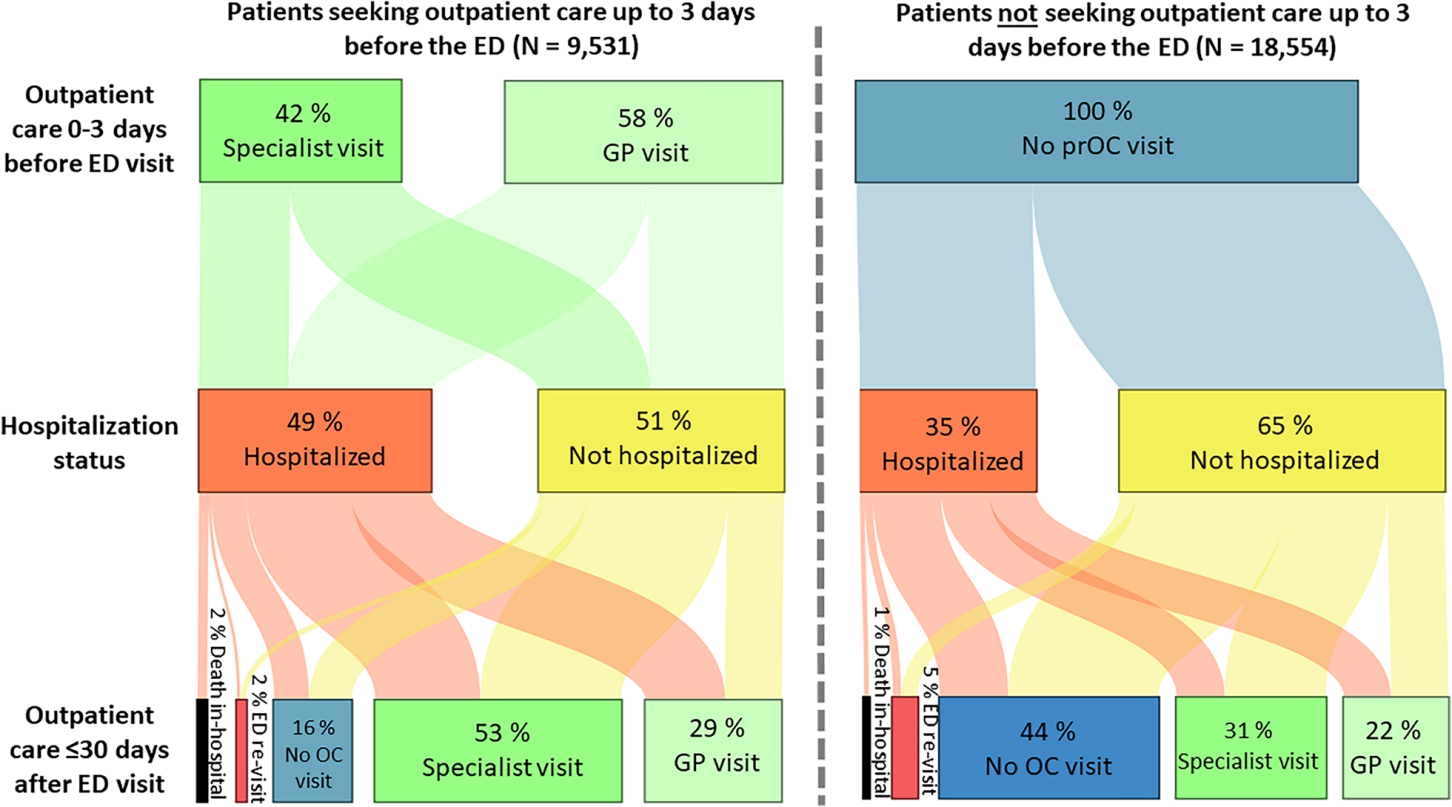
Treatment pathways of patients with AAP before and after their ED visit. The graph compares treatment pathways of 9531 patients who visited a general practitioner (including 235 patients for whom the type of OC was not documented) or specialist practice within 3 days before their ED visit (left Sankey diagram) versus 18,554 patients who did not (right Sankey diagram). *AAP* acute abdominal pain; *ED* emergency department; *GP* general practitioner; *OC* outpatient care; *prOC* prior outpatient care

Figure 5 show care trajectories of patients with and without prOC utilization. PrOC use was associated with hospitalization in 48.6% of the ED patients and followed by post-OC use in 82.1%. Patients who did not use prOC were less often hospitalized; 52.7% of them used post-OC within 30 days after the ED visit. The latter appeared to be independent of whether patients were hospitalized or not in both groups.

### Determinants for hospitalization and re-visits to the emergency department

In the multivariable logistic regression analysis, hospitalization after the ED was associated particularly with older age, and to a lesser extent with male sex, MTS category ‘urgent’, prOC use and ED district type (Table 2). As in bivariate analysis, prOC use strongly decreased the likelihood to re-visit the ED, independently of age, sex, MTS category, hospitalization, and ED district type (Table 2). In sensitivity analyses (adjusting for ED), the impact of MTS was increased for both endpoints (Hospitalization: 2.32; CI 2.17; 2.47. ED re-visit: 1.16; CI 1.01; 1.34) while the other results remained stable.

**Table 2:** Potential risk factors of hospitalization, and ED re-visits within 30 days

Independent variable	Endpoint: Hospitalization (n = 25,107; 89.4%)		Endpoint: ED re-visit within 30 days (n = 25,107; 89.4%)	
	Adjusted odds ratio	95% confidence interval	Adjusted odds ratio	95% confidence interval
Elderly (≥ 65 years vs. non-elderly)	3.05	2.87; 3.25	1.32	1.13; 1.55
Male (vs. female)	1.44	1.37; 1.52	0.96	0.84; 1.09
MTS status urgent (vs. non-urgent)	1.84	1.74; 1.95	1.10	0.96; 1.26
prOC use within 3 days before the ED (vs. non-prOC use)	1.71	1.61; 1.90	0.37	0.31; 0.44
Urban district ED type (vs. rural)	0.50	0.47; 054	1.08	0.92; 1.28
Hospitalized after the ED (vs. not)	n/i	n/i	0.78	0.67; 0.90

Results from multivariable logistic regression

*CI* confidence interval; *ED* emergency department; *MTS* Manchester Triage System; *n/I* not included; *prOC* prior outpatient care

## Discussion

### Main findings

This study provided an overview of patients with AAP in ED care in Germany regarding their characteristics, outpatient care utilization before and after the ED visit, hospitalization rate, and in-hospital mortality. The hospitalization rate in our study was 41.6% after the ED visit, with the most common diagnoses being diseases of the digestive or genitourinary system, or symptom-based diagnoses (R10). Approximately two thirds of the AAP patients did not seek prOC 3 days before attending the ED. Overall, almost 4% re-visited the ED within 30 days. The number of re-visit were significantly lower in patients who used OC 3 days before their ED visit compared to patients who did not (1.9% vs. 4.0%, respectively).

### Comparison with other studies

In line with other studies, the average AAP patient at the ED was 47.7 years old, female, and diagnosed with a symptomatic R10 diagnosis in the ED [1, 4, 12, 18]. An in-depth characterization by Pemmerl et al. (2021) of a comparatively smaller and younger AAP population in the ED of a German urban community hospital (N = 1,417) found a significantly higher hospitalization rate of 48.2% (*p* = 0.002) compared to our study. Yet, Helbig et al. [19]—whose study in two EDs in an urban area was similar in study design and inclusion criteria to our study—found a significantly lower hospitalization rate of 25.8% in 49,430 patients. Small single-center studies from Italy, Greece, and Poland showed also vastly differing hospitalization rates between 16.6 and 36.0% [1, 4, 6, 12, 14]. Two of these studies also reported ED re-visit rates up to 30 days post-ED which were significantly higher than in our study (6.5% and 10.9%, respectively; *p* < 0.001) [1, 6]. Differences in sample size, age, sex and urgency distribution, admission policies and remuneration-related incentives, the availability of hospital beds, as well as different study designs and small sample sizes may have contributed to these differences. We would argue that these differences underline the importance of large-scale studies, and that our study reports the most reliable data on hospitalization rates of patients with AAP to date.

### Relevance and outlook

The low hospitalization rate of patients who did not seek prOC up to 3 days before attending the ED (35.2%) may indicate that patients with low urgency experienced barriers to accessing prOC before coming to the ED [20]. In a sectoralized health care system such as that in Germany, the OC provider is meant to serve as a gate-keeper to primary and secondary care as well as to secure continuity and coordination of care [21]. In line with this expectation, we found that prOC use had—in addition to old age and urgent MTS status—considerable impact on hospitalization, pointing towards the role of prOC as a navigator for seeking acute care [22, 23]. Patients in Germany can effectively take the decision whether to seek care at the ED by themselves and often do not make use of the intended navigator role of a prOC referral [11, 24, 25]. Patients may opt to visit an ED for a specialist opinion or diagnostic imaging due to insufficient capacity of primary and secondary OC providers. Of the one third of patients who sought prOC before attending the ED in our study, the hospitalization rate was also unexpectedly low (48.6%). This may further indicate potential difficulties of OC resources to meet the complex clinical requirements or expectations of this patient population [20, 26, 27], in particular to provide diagnostic imaging procedures such as sonographies and CTs in a timely manner. Should health care systems aim to meet these requirements faster in OC to avoid overcrowded EDs? Some experts argue that for a complex medical condition such as AAP, the ED—not OC—is in fact the appropriate point of care, due to the immediate availability of laboratory services, imaging, monitoring and interdisciplinary treatment in one place [28]. Yet, most experts agree that it is necessary to alleviate pressure on EDs. Promising European approaches to do so aim towards improved coordination between urgent primary care and emergency care, namely the establishment of hospital-associated outpatient clinics at the hospital locations as well as adequate financial and structural recognition of the health care services that EDs provide [8, 29–32].

The role of prOC for hospitalizations, ED re-visits and OC use after the ED is unclear. It may be impacted by timely access to outpatient primary care including diagnostic imaging procedures, improved health literacy in patients, and discharge planning services for hospitalized patients [21, 23, 33–36]. Further investigations are needed, in particular with OC data that should be collected prospectively in European health care systems, to better determine the impact of these factors on the relationship between OC use and ED re-visits.

### Strengths and limitations

The present analyses were part of the INDEED study that collected data from 454,747 visits to 16 EDs. It is the largest study of ED patients in Germany to date. INDEED’s unique strength was that it linked ED data with outpatient care data on the individual patient level, which was unprecedented in Germany at this sample size. For Europe, the present analysis was the first evaluation of data from patients with AAP across treatment sectors including a large sample of 28,376 patients with 31,576 visits to 15 EDs. Another strength of our study was the retrospectively collected routine healthcare data of good quality, allowing insights into the real-world care of statutory health insurance companies’ patients.

However, our study had also some potential limitations. First, data availability and quality differed across EDs, making the data susceptible to systematic errors in documentation, availability bias, and diagnostic access bias. For example, documentation of medical treatment at the ED including recording of diagnostic procedures such as sonographies and computerized tomography is not standardized in Germany, is subject to limited or even restricted access, and was thus not reliably available for our study [37]. Further, referrals by OC providers could not be analyzed for our study since they are not part of the standardized routine data for EDs in Germany. A standardized documentation in the ED has been under discussion for years with no consensus yet reached. Similarly, ED diagnoses were not available or could not be retrieved for 13.9% of non-hospitalized ED patients in our study. We did not have the information weather a diagnosis was missing because it was not made, not documented or not retrieved during data extraction. The majority of diagnoses captured were ICD symptom diagnoses and conferred little of clinical relevance. Second, ED patients with specific diseases, amongst them diverticulitis or Morbus Crohn, may present with AAP but might not have been picked up by the inclusion criteria of this study depending on triage and diagnostic standards at the participating centers. Third, for patients who were included due to their ED diagnosis and for whom multiple ED diagnoses were documented, it was impossible to deduce if the AAP diagnosis was the leading diagnosis. Similarly, it could not be determined if AAP was the leading cause for prOC and post-OC visits. Fourth, for the outcomes ‘post-OC use’ and ‘revisit to ED’ within 30 days’ we did not focus only on AAP-related reasons but included all reasons for such visits and utilization. However, the results of a subgroup analysis indicated that this was unlikely to have influenced our results. Less than 1% of patients visited only physicians from specialties without relation to AAP, such as ophthalmologists. Still, we do not know if the OC physician referred the patients to the ED or if the decision to visit the ED was made independently by the patients. In addition, ED visits as well as hospitalizations may be underreported since patients may have visited EDs/hospitals that did not participate in the study. Fifth, our study included only patients with AAP who appeared at the ED (index patient) but not patients with AAP who were successfully treated by their GPs. Thus, our approach did not allow us to examine more comprehensively if going to a GP with AAP may affect hospitalization after ED visit or the revisit rate to ED.

## Conclusions

AAP is a leading cause for hospitalization of ED patients in Germany, with a large variety of underlying diagnoses and considerable in-hospital mortality. Our findings underline the clinical complexity of diagnosing and treating these conditions, not only but especially in the ED. Hospitalization after an ED visit with AAP was significantly more likely for the elderly, prOC users and men. Approximately one third of ED patients with AAP sought OC before and two thirds after attending the ED. The frequency of ED visits by AAP patients with prOC but without subsequent hospitalization may indicate difficulties of prOC resources to meet the complex diagnostic requirements and expectations of this patient population. A lower number of ED re-visits was associated with prOC use and age ≤ 65 years. Fewer ED re-visits in prOC users indicate effective care in this subgroup. The interaction between ED and prOC services of patients with AAP needs to be further investigated including prospective studies with primary data collection to consider more confounding factors related to comorbidities and lifestyle, including substance abuse.

### Supplementary Information

Additional file 1.

## Data Availability

The data that support the findings of this study are not publicly available due to the high sensitivity of clinical data of the patients treated in the emergency department.

## Abbreviations

AAP: Acute abdominal pain
BBSR: German Federal Office for Building and Regional Planning
CI: Confidence intervals
CT: Computed tomography
ED: Emergency department
GP: General practitioner
ICD: International Classification of Diseases
K35: Acute appendicitis
K56: Paralytic ileus and intestinal obstruction without hernia
K57: Diverticular disease of intestine
K80: Cholelithiasis
K85: Acute pancreatitis
MTS: Manchester triage system
N10: Acute tubulo-interstitial nephritis
N13: Obstructive and reflux uropathy
OC: Outpatient care
OR: Odds ratio
post-OC: Post-outpatient care up to 30 days after ED visit
prOC: Outpatient care up to 3 days prior to the ED visit
R10: Abdominal and pelvic pain
SD: Standard deviation
