# Supplementary material for: Health care utilization of patients with acute abdominal pain before and after emergency department visits
Source: Scand J Trauma Resusc Emerg Med. 2024 Aug 12;32:68. doi: 10.1186/s13049-024-01237-7 (PMC11320862; doi:10.1186/s13049-024-01237-7)
Supplement: Supplementary file 1 — Additional file 1. [file 13049_2024_1237_MOESM1_ESM.docx]

# Sample characteristics and treatment pathways before and after the ED visit, by age group and sex

|  | **Age group** |  | **Sex** |  |
| --- | --- | --- | --- | --- |
|  | **Elderly [65+ years]**  **(N=6721; 23.9%)** | **Non-elderly**  **[<65 years]**  **(N=21364; 76.1%)** | **Men**  **(N=11710; 41.7%)** | **Women**  **(N=16375; 58.3%)** |
| Age, mean (SD) | 76.5 (7.4) | 38.7 (12.7) | 50.1 (19.2) | 46.0 (20.2) |
| Female, N (%) | 3587 (53.4) | 12788 (59.7) | 0 (0) | 16375 (100) |
| Urban area, N (%) | 4911 (73.1) | 17493 (81.9) | 9297 (79.4) | 13107 (80.0) |
| MTS status “urgent”, N (%) | 3911 (58.2) | 11994 (56.1) | 6772 (57.8) | 9133 (55.8) |
| Missing, N (%) | 771 (11.5) | 2207 (10.3) | 1258 (10.7) | 1720 (10.5) |
| prOC use, N (%) | 2959 (44.0) | 6572 (30.8) | 3728 (31.8) | 5803 (35.4) |
| Type of prOC provider, N (%) |  |  |  |  |
| General Practitioner | 1725 (265.7) | 3568 (16.7) | 2294 (19.6) | 2999 (18.3) |
| Specialist | 1164 (17.3) | 2839 (13.3) | 1334 (11.4) | 2669 (16.3) |
| Not documented | 70 (1.0) | 165 (0.8) | 100 (0.9) | 135 (0.8) |
| Hospitalized, N (% of total N) | 4175 (62.1) | 6991 (32.7) | 5243 (44.8) | 5923 (36.2) |
| ICD-10 hospital diagnoses |  |  |  |  |
| Missing, N (% of hospitalized) | 35 (0.8) | 98 (1.4) | 60 (1.1) | 73 (1.2) |
| Top1 (% of total) | K56 (4.8) | R10 (3.4) | R10 (3.2) | R10 (3.4) |
| Top2 (% of total) | K80 (4.3) | K35 (2.9) | K35 (3.1) | K80 (2.6) |
| Top3 (% of total) | R10 (3.3) | K80 (2.0) | K85 (2.6) | K35 (2.0) |
| Top4 (% of total) | K57 (3.1) | K85 (1.5) | K80 (2.5) | K56 (1.8) |
| Top5 (% of total) | K85 (2.6) | N13 (1.4) | K56 (2.4) | K57 (1.7) |
| In-hospital mortality N (% of hospitalized) |  |  |  |  |
| Death reported | 256 (6.1) | 93 (1.3) | 173 (3.3) | 176 (3.0) |
| Missing | 118 (2.8) | 412 (5.9) | 207 (4.0) | 323 (5.5) |
| Post-OC use, N (%) | 4670 (69.5) | 12933 (60.5) | 6773 (57.8) | 10830 (66.1) |
| Type of post-OC provider, N (%) |  |  |  |  |
| General Practitioner | 1937 (28.8) | 4862 (22.8) | 3176 (27.1) | 3623 (22.1) |
| Specialist | 2727 (40.6) | 8054 (37.7) | 3587 (30.6) | 7194 (43.9) |
| Not documented | 6 (0.1) | 17 (0.1) | 10 (0.1) | 13 (0.1) |
| Post-OC use after hospital, N (% of hospitalized) | 2884 (69.1) | 4567 (65.3) | 3346 (63.8) | 4105 (69.3) |
| Type of post-OC provider, N (%) |  |  |  |  |
| General Practitioner | 1341 (32.1) | 2107 (30.1) | 1707 (32.6) | 1741 (29.4) |
| Specialist | 1537 (36.8) | 2452 (35.1) | 1633 (31.2) | 2356 (39.8) |
| Not documented | 6 (0.1) | 8 (0.1) | 6 (0.1) | 8 (0.1) |
| ED re-visit in 30 days | 280 (4.2) | 821 (3.8) | 450 (3.8) | 651 (4.0) |

Abbreviations: A09, Other gastroenteritis and colitis of infectious and unspecified origin; ED, Emergency department; ICD, International classification of disease; MTS, Manchester Triage System; N, Number; N10, Acute tubulo-interstitial nephritis; N13, Obstructive and reflux uropathy; OC, Outpatient care; post-OC, post-outpatient care (up to 30 days after ED visit); prOC, prior outpatient care (up to 3 days before ED visit); R10, Abdominal and pelvic pain; K35, Acute appendicitis; K56, Paralytic ileus and intestinal obstruction without hernia; K57, Diverticular disease of intestine; K80, Cholelithiasis; K85, Acute pancreatitis.
